# Chromium Morpho-Phytotoxicity

**DOI:** 10.3390/plants9050564

**Published:** 2020-04-29

**Authors:** Abdul Wakeel, Ming Xu

**Affiliations:** Key Laboratory of Geospatial Technology for the Middle and Lower Yellow River Regions, College of Environment and Planning, Henan University, Kaifeng 475004, China

**Keywords:** chromium, plant, biomass, growth retardation, nutrients uptake

## Abstract

Chromium (Cr) is considered as one of the chronic pollutants that cause damage to all living forms, including plants. Various industries release an excessive amount of Cr into the environment. The increasing accumulation of Cr in agricultural land causes a significant decrease in the yield and quality of economically important crops. The Cr-induced biochemical, molecule, cytotoxic, genotoxic, and hormonal impairments cause the inhibition of plant growth and development. In the current study, we reviewed Cr morpho-phytotoxicity related scientific reports published between 2009 to 2019. We mainly focused on the Cr-induced inhibition of seed germination and total biomass production. Furthermore, Cr-mediated reduction in the root, branches, and leave growth and development were separately discussed. The Cr uptake mechanism and interference with the macro and micro-nutrient uptake were also discussed and visualized via a functional model. Moreover, a comprehensive functional model has been presented for the Cr release from the industries, its accumulation in the agricultural land, and ultimate morpho-phytotoxicity. It is concluded that Cr-reduces plant growth and development via its excess accumulation in the plant different parts and/or disruption of nutrient uptake.

## 1. Introduction

Chromium (Cr) is considered one of the major carcinogens, and is categorized 7th among the top 20 hazardous pollutants by the Environmental Protection Agency, United States of America (EPA, US) [1,2,3]. Cr(VI) and Cr(III) are the most stable form of Cr in the environment. On the bases of bioavailability in soil and translocation to different plant parts, Cr(VI) is reported to be more toxic than Cr(III) [3,4,5]. The industrial process coupled with anthropogenic and natural processes have resulted in increased accumulation of Cr in both terrestrial and aquatic ecosystems [3,4,6]. Chromium in soil and water directly affects human, animal, and plant physiology, and may accumulate within food chains, which can be a serious health threat to the secondary (herbivores) and tertiary (carnivores and omnivores) consumers [3,7,8].

Various physiological factors including plant species, rate and types of root secretion, the surface area of the root, and transpiration rate regulate the absorption, translocation, and accumulation of the Cr in plants [9,10]. Chromium mainly accumulates in the plant roots that triggers the uptake and translocation of Cr to the aerial plant parts [11,12,13]. The toxic Cr level can provoke various morphological, physiological, biochemical, and molecular alterations in plants [14,15].

The toxic level of Cr inhibits plant growth and development, induces ultrastructural changes in subcellular compartments (cell wall, cell membrane, plastids, chloroplast, mitochondria, Golgi bodies, endoplasmic reticulum, vacuole, nuclei, and microtubules), persuades leaves chlorosis, root cell damage, reduces total pigment contents, disturbs water and mineral nutrition balance, alters enzymatic activities, and modulates cell division and cell cycle [3,16,17,18,19,20].

**Figure 1:**
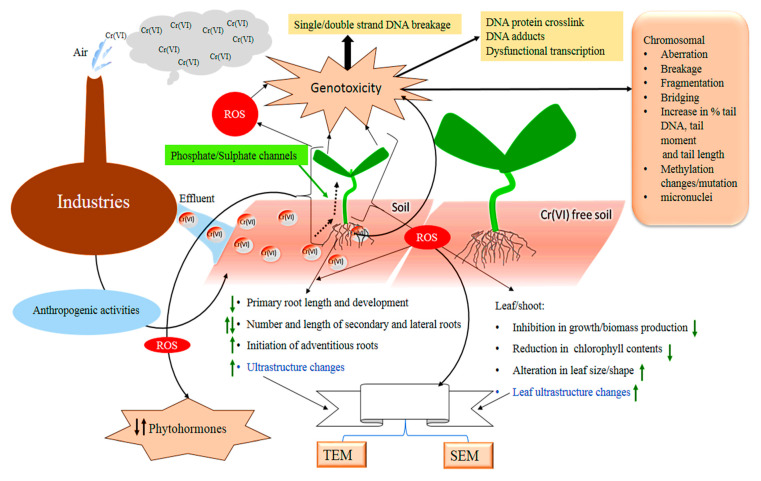
A functional model for the release, accumulation, and toxicity in plants. Cr is released from/through the industrial processes and anthropogenic activities in the soil. The model also visualizes the uptake of Cr by the plant roots, translocation to the shoots. The Cr-induced morphological, physiological, biochemical, molecular, hormonal, and ultrastructural changes in plants are also summarized in the model.

The process of increasing Cr accumulation in soil, its uptake/translocation in plants, Cr-induced morpho-physiological, biochemical, molecular, ultrastructural, and hormonal changes in plants are summarized and visualized in (Figure 1). In the current study, we reviewed the most recent studies regarding Cr-induced inhibition in seed germination and growth retardation in roots, branches, leaves, and total biomass in various plant species.

## 2. Chromium-Mediated Control of Seed Germination

The first phenotypic and physiological change mediated by Cr in plants is seed germination, which is very important for the continuity of the plant life cycle [21]. Endogenous and exogenous stimuli mediated genetic and epigenetic changes were reported to be involved in the regulation of seed germination, and plant biochemical, molecular and ultrastructural changes [21,22,23]. Chromium-induced inhibition of seed germination in various plant species have been reported, and the germination rate depends on Cr(VI) concentration and type of plant species as shown in (Table 1). Chromium stress affects the activities of both alpha and beta-amylase, which are the sources of energy provided to the emerging embryos. In summary, Cr reduces the activity of amylase, leading to the reduced sugar availability for energy production, and inhibits the rate of plant seed germination [24].

## 3. Chromium-Induced Modulation of the Root Growth and Development

The plant root is the first organ that encounters soil pollutants, Cr is one of the most important soil pollutants, which affects root growth and development [14,23]. Chromium-induced reduction in the root growth mainly depends on the plant species, Cr-type and its concentration as shown in the (Table 2) Chromium is also involved in the regulations of secondary root growth and number, lateral root development, root hair, and formation of adventitious roots [20,24,33]. The reduced root length with a brownish appearance and reduced root hair number have been observed in *Zea mays*, exposed to high Cr(VI) levels [33]. The root growth inhibition mediated by Cr(VI), maybe due to the inhibition of cell division and reduction in the cell size of the elongation zone [14]. The reductions of mitotic cell division in *Amaranthus viridis* and *Arabidopsis thaliana*, have been reported, which is associated with the reduction in cell cycle-related genes and alterations in the cellular ultrastructure [3,14].

## 4. Chromium-Induced Alteration in the Shoot Growth and Development

The growth and development of the plants’ shoots are greatly compromised by the exposure to high Cr-concentrations and the degree of toxicity depends on the plant species, Cr-type, and concentration [3,4]. The Cr-induced alterations in various plant species are shown in the (Table 3). In a recent study, 32 plant species were exposed to 1000 mg/kg Cr(VI), they found that Cr(VI)-reduced the stem growth of 94% species [39]. Chromium-induced stem growth inhibition maybe due to the Cr-induced damages in the roots, which make it incapable of sufficient nutrients and water uptake [3,4]. Furthermore, the transport and accumulation of toxic Cr-level may have a direct inhibitory as well as structural and ultrastructural damaging effects on the shoot growth, development, and the capability of performing certain physiological, biochemical, molecular, and metabolic activities [3].

## 5. Chromium Mediated Changes in Leaf Growth and Morphology

Leaf structure and growth have been intensely investigated as an important indicator under various abiotic stresses [42]. Chromium-induced various biochemical, ultrastructural, and physiological changes have also been reported [19]. The leaf morphological changes in Cr-treated seedlings indicated that the appearance of the leaf was significantly changed in the size, and it was chlorotic and wilted as compared to those plants exposed to control condition [39,43]. The prolonged Cr exposure caused permanent necrosis, turned wilted and dry, and finally shed of leaves has been reported in the Cr-treated plants [44]. The reduction in leaf size of Arabidopsis thaliana upon Cr exposure is also reported, which can be due to the inhibition in cell division [15]. The watermelon plants exposed to Cr toxicity showed a phenotype of reduced number and size of leaves with a yellow appearance, wilted and turgor loss due to low water contents in the leaves [45]. Chromium-induced phenotypic alteration and growth inhibition in the leaf of various plant species have been summarized in the current review as shown in (Table 4).

## 6. Chromium-Mediated Changes in Total Biomass Production in Plants

The biomass production is considered proportional to yield, which is greatly compromised in the plants exposed to Cr, indicating that Cr is reducing plant biomass as well as the yield of the important crops worldwide [15,19,47,48]. Numerous, species were investigated and reported to exhibit reduced biomass production under high Cr(VI) levels, and the toxicity varies based on the different plant species, and concentration and type of Cr(VI) used as shown in (Table 5). Several factors such as reduction/imbalance in the uptake/translocation of water and nutrients, cell division and division rate inhibition, selective inorganic nutrient uptake inefficiency, increased ROS accumulation, essential nutrient substitution from ligand and plant key molecules, and Cr-induced ROS mediated alteration and damages to plastids, pigment contents, mitochondria, lipids, RNA, and DNA are involved in the Cr-decreased growth, development, and yield in plants at molecular, cellular, tissue, and organ levels are involved in the alteration in the plant biomass production [3,15,16,17,19,47,49,50]. The degree of severity of these factors depends on the type of Cr and plant species [3]. The hyper heavy metal accumulator plants such as *Brassica juncea* and *Alyssum maritime* are were reported to be potentially more tolerant and can survive a range of high Cr concentrations [4,9].

## 7. Chromium Interferes with the Uptake and Translocation of Macro and Micronutrients

Chromium interferes with the nutrients uptake and translocation mechanisms of plants due to the structural similarity with the various essential ions [58,59]. The interference of Cr with the uptake and translocation of macro and micronutrients depends on the type of plant species and Cr-type. The decrease in the common nutrient uptake/translocation could be because of the competitive binding potential of Cr with carrier channels and reduced plasma membrane H^+^ATPase activity [3]. Chromium exposure may displace the nutrients from the binding sites both in the soil and inside the plant body. Mostly, Cr is reported for playing an antagonistic role in the uptake and translocation of essential nutrients, it also interacts synergistically with some essential nutrients such as Cu, Ca, Mg, and Mn [60,61]. The Cr-induced interruptions and variations in the nutrients uptake and translocation have been reviewed in (Table 6).

## 8. Conclusions

**Figure 2:**
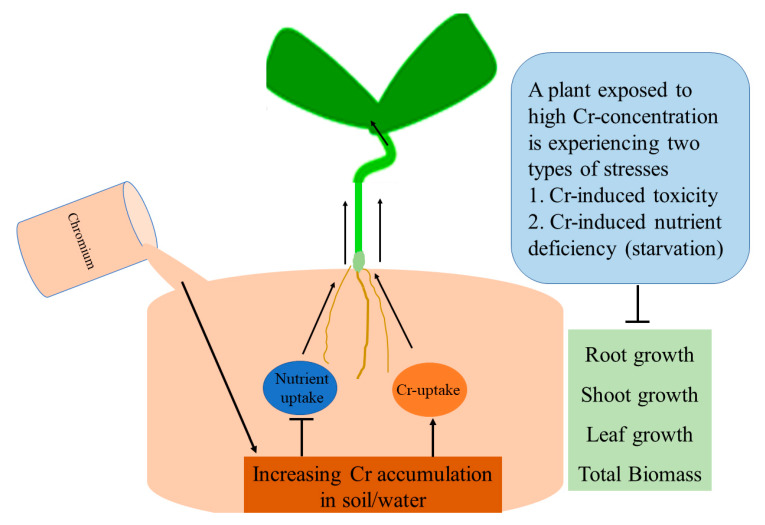
Functional model for the Cr-induced nutrient uptake inhibition and plant growth retardation. Increasing concentration of Cr in soil/water compete with the nutrients uptake that is leading in the increasing Cr accumulation in plant and reduction of nutrients. So, in the presence of high Cr concentration plant faces two stress simultaneously: 1. High Cr accumulation-mediated phytotoxicity. 2. Nutrient deficiency-mediated metabolic abnormality.

Based on the available literature reviewed in the current study, we can conclude that increasing Cr concentration reduces plant biomass accumulation. The plants have no specialized intake channels for the Cr uptake. Cr competes with essential elements (macro and micro) for access to plant uptake machinery. High Cr concentration reduces the uptake of essential elements and increases its accumulation in the plant in different parts, which causes various phenotypic, ultrastructural, and biochemical changes in plants. Cr-induces endogenous plant stress molecules that may cause a reduction in plant growth and biomass accumulation. The reduction in the essential element may also participate in the retardation of plant growth and biomass production (Figure 2).

## Figures and Tables

**Table 1:** Chromium-induced seed germination inhibition in various plant species.

Plant Species	Common Name	Chromium Concentration	Medium	Time of Exposure (Days)	Seed Germination (%)	References
***Avena sativa***	Oat	500 mg/kg Cr(VI) 2000 mg/kg Cr(III)	Soil	7	≈82 ≈95	[25]
***Beta vulgaris***	Swiss chard	50 µM Cr(III)	Distilled water	12	71	[26]
***Brassica juncea***	Mustard	300 µM Cr(VI)	½-strength Hoagland	3	80.8	[27]
***Brassica oleracea***	Cabbage	300 mg/kg Cr(VI)	Distilled water	3	≈65	[28]
***Cajanus cajan***	Pigeon Pea	100 ppm	Distilled water	3	93	[29]
***Cucumis sativus***	Cucumber	300 mg/kg Cr(VI)	Distilled water	3	≈96	[28]
***Glycine max***	Soybean	200 mg/L Cr(VI)	Hydroponic	-	72.6	[30]
***Lactuca sativa***	Lettuce	300 mg/kg Cr(VI)	Distilled water	3	≈50	[28]
***Lactuca sativa***	Lettuce	50 µM Cr(III)	Distilled water	12	94	[26]
***Oryza sativa***	Rice	100 µM Cr(VI)	Distilled water	4	≈50	[31]
***Sorghum bicolor***	Sorghum	500 mg/kg Cr(VI) 2000 mg/kg Cr(III)	Soil	7	≈60 ≈10	[25]
***Spinacia oleracea***	Spinach	50 µM Cr(III)	Distilled water	15	64	[26]
***Triticum aestivum***	Wheat	100 ppm 300 mg/kg Cr(VI) 500 mg/kg Cr(VI) 2000 mg/kg Cr(III)	Distilled water Distilled water Soil	0.17 3 7	63 ≈90 ≈70 ≈25	[32] [28] [25]
***Zea mays***	Corn	300 mg/kg Cr(VI)	Distilled water	3	≈99	[28]

**Table 2:** Chromium-induced reduction in root growth as compared to control of various plant species.

Plant Species	Common Name	Chromium Concentration	Medium	Time of Exposure (Days)	Root Growth (%)	References
***Arabidopsis thaliana***	Arabidopsis	200 µM Cr(VI)	½ MS	1	92.8	[14]
***Avena sativa***	Oat	500 mg/kg Cr(VI) 2000 mg/kg Cr(III)	Soil	7	≈40 ≈55	[25]
***Brassica campestris***	Cabbage	1 mg/L Cr(VI)	½-strength Hoagland	21	≈35 FW	[34]
***Brassica juncea***	Mustard	300 µM Cr(VI)	½-strength Hoagland	15	43.7	[27]
***Brassica napus***	Oilseed Rape	400 μM Cr(VI)	Hoagland’s	6	≈50	[35]
***Brassica oleracea***	Cabbage	300 mg/kg Cr(VI)	Distilled water	3	≈25	[28]
***Cajanus cajan***	Pigeon Pea	100 ppm	Distilled water	10	32	[29]
***Cucumis sativus***	Cucumber	300 mg/kg Cr(VI)	Distilled water	3	≈15	[28]
***Lactuca sativa***	Lettuce	300 mg/kg Cr(VI)	Distilled water	3	<10	[28]
***Oryza sativa***	Rice	80 µM Cr(VI)	¼ -strength Kimura B	7	78	[36]
***Sorghum bicolor***	Sorghum	500 mg/kg Cr(VI) 2000 mg/kg Cr(III)	Soil	7	≈10 ≈30	[25]
***Triticum aestivum***	Wheat	500 µM Cr(VI) 10 mg/kg Cr(VI) 300 mg/kg Cr(VI) 500 mg/kg Cr(VI) 2000 mg/kg Cr(III)	Sand Quartz sand Distilled water Soil	- 7 3 7	≈57 ≈20 < 10 ≈10 ≈45	[37] [38] [28] [25]
***Zea mays***	Corn	300 mg/kg Cr(VI) 173 µM Cr(VI)	Distilled water Hydroponic	3 7	≈43% ≈70%	[28] [33]

**Table 3:** Chromium-reduced shoot growth as compared to control in various plant species.

Plant Species	Common Name	Chromium Concentration	Medium	Time of Exposure (Days)	Shoot Growth (%)	References
***Arabidopsis thaliana***	Arabidopsis	800 µM Cr(VI)	½-strength MS	2	≈50 FW	[15]
***Avena sativa***	Oat	500 mg/kg Cr(VI) 2000 mg/kg Cr(III)	Soil	7	Reduced	[25]
***Brassica campestris***	Cabbage	1 mg/L Cr(VI)	½-strength Hoagland	21	≈70 FW	[34]
***Brassica juncea***	Mustard	300 µM Cr(VI)	½-strength Hoagland	15	89.1	[27]
***Brassica napus***	Oilseed Rape	400 μM Cr(VI)	Hoagland	6	58–67	[35]
***Cajanus cajan***	Pigeon Pea	100 ppm	Distilled water	10	Reduced	[29]
***Hordeum vulgare***	Barley	100 μM Cr(VI)	Nutrient solution	50	≈7–20 DW	[40]
***Oryza sativa***	Rice	80 µM Cr(VI)	Hydroponic	7	77	[36]
***Parthenium hysterophorus Solanum nigrum***	Santa Maria Black Nightshade	500 µM Cr(VI)	Soil	21	43 FW 65 DW 110 FW 115 DW	[41]
***Sorghum bicolor***	Sorghum	500 mg/kg Cr(VI) 2000 mg/kg Cr(III)	Soil	7	Reduced	[25]
***Triticum aestivum***	Wheat	500 µM Cr(VI) 10 mg/kg Cr(VI)	Sand Quartz sand	7	≈80% ≈80%	[37] [38]
***Zea mays***	Corn	173 µM Cr(VI)	Hydroponic	7	≈80%	[33]

**Table 4:** Chromium-altered leaf morphology and growth as compared to control in various plant species.

Plant Species	Common Name	Chromium Concentration	Medium	Time of Exposure (Days)	Induced Changes in Leaf Growth and Morphology	References
***Arabidopsis thaliana***	Arabidopsis	800 μM Cr(VI)	½-strength MS	2	Reduced: growth, water content (RWC), chlorophyll (chl), cell and tissue viability	[15]
***Brassica juncea***	Mustard	300 μM Cr(VI)	Semi-hydroponic medium	5	Reduced: growth, RWC, and chl content	[46]
***Brassica napus***	Oilseed Rape	400 μM Cr(VI)	Hoagland	6	61%–71% Reduced biomass	[35]
***Hordeum vulgare***	Barley	100 μM Cr(VI)	Nutrient solution	50	≈62%–67% Reduced DW	[40]
***Oryza sativa***	Rice	80 µM Cr(VI)	Hydroponic	7	Chlorosis	[36]
***Zea mays***	Corn	173 µM Cr(VI)	Hydroponic	7	Reduced leaf number	[33]

**Table 5:** Chromium-meditated reduction in the total plant biomass as compared to control in various plant species.

Plant Species	Common Name	Chromium Concentration	Medium	Time of Exposure (Days)	Total Biomass Production (%)	References
***Amaranthus viridis and Amaranthus cruentus***	Green and Blood Amaranth	50 μM	½-strength Hoagland	7	> 50 FW ≈80 FW	[51]
***Arabidopsis thaliana***	Arabidopsis	800 μM Cr(VI)	½-strength MS	2	50 FW 75 DW	[15]
***Brassica juncea***	Mustard	300 μM Cr(VI)	Semi-hydroponic medium	5	80–89 growth	[46]
***Brassica juncea***	Mustard	100 µM Cr(VI)	Soil	20	> 50 FW and DW	[52]
***Brassica napus***	Oilseed Rape	400 μM Cr(VI)	Hoagland	6	67 DW	[35]
***Brassica napus***	Rapeseed	500 μM Cr	Soil	56	30.6 FW 28 DW	[53]
***Citrus reticulata***	Kinnow Mandarin	750 μM Cr(VI)	Soil	120	63 DW	[54]
***Cyperus alternifolius and Coix lacryma-jobi***	Umbrella Palm and Adlay Millet	40 mg/L Cr(VI)	Soil	120	77 DW 44 DW	[55]
***Hordeum vulgare***	Barley	100 μM Cr(VI)	Quartz sand	60	≈23.7DW	[56]
***Lemna minor***	Duckweed	500 μM Cr(VI)	SIS growth medium	7	60	[57]
***Oryza sativa***	Rice	80 µM Cr(VI)	Hydroponic	7	58	[36]
***Parthenium hysterophorus Solanum nigrum***	Santa Maria Black Nightshade	500 µM Cr(VI)	Soil	21	65.5 FW 64.DW 110 FW 106 DW	[41]
***Solanum melongena***	Eggplant	25 µM Cr(VI)	½-strength Hoagland	7	87 FW 83 DW	[48]
***Triticum aestivum***	Wheat	500 µM Cr(VI)	Sand Quartz sand	7	≈65%	[37]
***Zea mays***	Corn	173 µM Cr(VI)	Hydroponic	7	≈85 FW	[33]

**Table 6:** Chromium-induced alteration in the uptake and translocation of the essential nutrients in various plant species.

Plant Species	Common Name	Nutrients	Alteration in Uptake/Translocation	Reference
***Brassica juncea***	Brown Mustard	Na, K, Ca, Mg, C, H, and N	Reduced both	[27]
***Cocos mucifera***	Coconut Palm	Fe, K, Cu, Zn, Mn, and Mg	Uptake	[3]
***Hordeum vulgare***	Barley	P, K, Mg, S, Fe, Zn, Mn, and Ca	Uptake and Translocation	[40]
***Lactuca sativa***	Lettuce	K, Mg, Fe, and Zn	Uptake/translocation	[62]
***Oryza sativa***	Rice	N, P, K, Ca, Mg, Mn, Zn, Fe, and Cu	Uptake/translocation	[63,64]
***Pisum sativum***	Pea	Decreased micro and macronutrients (except S)	Uptake/translocation	[65]
***Raphanus sativus***	Radish	Fe, S, P, Zn, Mn, Cu, and B	Translocation	[59]
***Solanum lycopersicum and Solanum melongena***	Tomato and Eggplant	Affected N, P and K content	Translocation	[66]
